# Engagement by New South Wales Marine Estate Users with and Evaluation of Communication Approaches to Strengthen Biosecurity Practices

**DOI:** 10.1007/s00267-025-02221-2

**Published:** 2025-07-08

**Authors:** Jennifer Manyweathers, Lynne Hayes, Ben Rampano, Nat Higgins, Marta Hernandez-Jover

**Affiliations:** 1 School of Agricultural, Environmental and Veterinary Sciences, Charles Sturt University, Wagga Wagga, NSW Australia; 2 Gulbali Institute, Charles Sturt University, Wagga Wagga, NSW Australia; 3 NSW Department of Primary Industries & Regional Development, Port Stephens Fisheries Institute, Port Stephens, NSW Australia

**Keywords:** Marine biosecurity, New South Wales marine estate, Pests and diseases, Recreational vessels, Recreational boats, Communication interventions, Social media

## Abstract

Marine environments are susceptible to the impact of human boating activities that facilitate incursions of marine pests and diseases, including invasive alien species (IAS). Movement and maintenance of recreational boats is largely unregulated, and if boats are improperly maintained, movement of IAS on these unmaintained boats can impact livelihoods and food security. This project evaluated the impact of communication and engagement interventions implemented within the New South Wales marine estate that aimed at strengthening biosecurity practices of small to medium permanently moored boat owners/managers. A mixed method approach using a cross-sectional survey and interviews was used to evaluate exposure to and impact of the communication and engagement strategies, including Facebook images and videos on biosecurity practices in the NSW marine estate. Participants expressed a desire for information about marine pests and diseases via electronic newsletters (43.3%, *n* = 58) and of the survey participants who used Facebook, the majority (90.2%, *n* = 119) had not seen the communication interventions. Interview participants identified some infrastructural barriers to keeping their boats clear of biofouling despite a desire to do so. Overall, most participants showed awareness of and wanted to help protect waterway health, which supports ongoing efforts by New South Wales Department of Primary Industries Agriculture and Biosecurity Aquatic unit to improve biosecurity practices of all marine estate stakeholders and informs future development of aquatic biosecurity communication and engagement strategies.

## Introduction

The interconnectedness of waterways means that aquatic environments are susceptible to the impact of human boating activity that facilitate the spread of IAS, accompanied by limited research looking at marine ecosystems and the impact of pests and diseases contributing to the risk (Ojaveer et al. 2015). Recreational boats contribute to the secondary dissemination of pests and diseases from trade pathways to smaller bodies of water (Zabin 2014; Seebens et al. 2016), and are largely unhindered by regulations that guide biofouling management on commercial boats (Lane et al. 2018). with global marine pest and disease incursions predicted to increase over the next 10 years (Sardain et al. 2019).

In the New South Wales (NSW, Australia) marine estate, movement of small to medium recreational boats with unmanaged biofouling, the accumulation of aquatic plants, animals and microorganisms on and within boats (International Maritime Organization 2013), is a significant biosecurity threat to marine and aquatic industries, the environment and communities that rely on healthy waterways (Raftos et al. 2014). Marine pests can attach to boat hulls, niche areas such as internal water systems, including ballast (including water and heavy materials), and intake pipes, rudder, propeller and anchors/chain cabinets and equipment (Clarke Murray et al. 2011; Lane et al. 2018). Biofouling is particularly problematic in permanently moored boats, as opposed to those boats that are removed, cleaned and stored on trailers after use, with a strong positive relationship between the number of days since cleaning and an increase in biofouling biomass (Lane et al. 2018).

Within the NSW marine estate, evidence suggests that stakeholders, including boat owners and service providers, may not have a good understanding of biofouling and pest and disease translocation and the effect of aquatic pests and diseases on the marine estate, with just over one third of participants in a 2017 Australian recreational boat owners’ survey reporting that they did not properly dispose of biofouling waste properly and just under 10% unsure of where the waste ended up. (Stenekes et al. 2018).

This is of particular relevance given that these stakeholders are crucial to the safeguarding of marine biosecurity (Lott and Rose 2016; unpublished results). Evidence suggests that awareness of biofouling alone is insufficient to support adoption of biosecurity practices (Golebie et al. 2021b; Payne et al. 2023). Instead, a deeper understanding of stakeholder perspective, sense of agency, perception of risk, social networks and collective action and their impact on long term behaviour adoption is needed (Polonsky et al. 2004; Golebie et al. 2023; Maru et al. 2025).

To better understand the baseline awareness and practices, risk perception and beliefs, guided by the COM-B model of behaviour change (Michie et al. 2011; Hine et al. 2020), Phase 1 of this project used a cross-sectional survey of small to medium permanently moored boat owners in NSW. The survey was undertaken in 2021 by Charles Sturt University researchers and NSW Department of Primary Industries (DPI; which became the NSW Department of Primary Industries & Regional Development from 1 July 2024) Agriculture and Biosecurity Aquatic unit. Findings included a general perception by participating boat owners that the government had not demonstrated appropriate responsibility for marine biosecurity and that communication approaches were inadequate (unpublished results) This identification of the ‘other’ when allocating responsibility is not unique to the Australian or marine environments (Manyweathers et al. 2017; Golebie et al. 2021a), and was instrumental in developing recommendations from Phase 1 that included investment in strengthening partnerships with marine stakeholders and enhancing communication and engagement pathways (unpublished results). Between 2022–2024, communication and engagement interventions were implemented, including employment of staff dedicated to biosecurity engagement, and a social media campaign aimed at increasing awareness of reporting unusual pests on biofouling.

The current mixed methods study (Phase 2) covered in this paper re-evaluated biosecurity practices and awareness, and explored interactions by boat owners with and impact of the communication and engagement interventions, in particular, exposure to and impact of Facebook biosecurity messages posted as images and videos by NSW DPI, and self-reported changes in biosecurity practices in 2023–24.

## Materials and Methods

A mixed methods approach, using an online questionnaire and interviews, was used to capture any self-reported changes by NSW boat owners, in behaviour and engagement with the NSW DPI marine biosecurity engagement interventions. Descriptive analysis of the survey findings and interview responses were used to consider the impact of the interventions and to inform future approaches to strengthening relationships between marine estate stakeholders and to decrease the biosecurity risk faced by the NSW marine estate from pest and disease incursions.

All research activities were approved by the Human Research Ethics Committee at Charles Sturt University on 2^nd^ April 2024 (H24021).

### Questionnaire Development, Distribution and Analysis

A cross sectional survey, using a questionnaire of 18 open and closed questions was developed for the purpose of collecting the data from NSW boat owners across the following areas: demographics (9), current biosecurity knowledge and practices (3), interactions with and impacts of the communication and engagement interventions implemented by NSW DPI (3), and preferred communication sources and approaches (3). The questionnaire can be found in Supplementary Material.

The questions were entered onto an online survey platform (Survey Monkey Inc, San Mateo, California, USA, www.surveymonkey.com) for data collection. The target population for the survey was owners (or those who manage) small to medium (less than 30 metres) boats that are permanently moored or at a berth in coastal NSW waters.

The questionnaire was reviewed by NSW DPI with feedback incorporated, and piloted by two boat owners, members of the NSW Marine Pest Working Group.

The questionnaire was open between 7th June and 12th August 2024. The NSW DPI Aquatic Biosecurity and Communications teams distributed the survey invitation to all relevant stakeholders via email, websites, and social media. Websites included NSW DPI, NSW DPI Marine Pests, NSW DPI Fisheries, Afloat Magazine website, the Marina Industry Association, and Boating Industry Association. Social media invitations were posted and boosted on Facebook pages including NSW DPI Animal Biosecurity, NSW Marine Estate, Fisheries, Workplace and Local Land Services, and Transport NSW (NSW Maritime). The invitation was also placed on Service NSW Twitter, the NSW Marine Estate Instagram, the NSW Game Fishing Club social media, NSW Marine Pest Working Group partner agencies intranet and all Afloat Magazine social media channels. Stakeholders from Phase 1 of the study were also invited to distribute the questionnaire through their networks, with two marinas agreeing to assist.

Survey participants were then given the opportunity to express their interest in participating in the follow up interview with full anonymity of the survey responses.

Data were downloaded from the survey platform to Microsoft® Excel® (Microsoft 365 MSO (Version 2402 Build 16.0.17328.20346) 32-bit) and cleaned. Descriptive statistics were analysed using SPSS (IBM Corp. Released 2024, Version 29.0).

### Development, Recruitment, Implementation and Analysis of Stakeholder Interviews

The interviews focussed on self-reported engagement with the communication and engagement interventions undertaken by NSW DPI, and included questions about communication networks and information sources, and behaviours and attitudes related to hull cleaning and bio fouling more generally. The research team developed the initial interview guide, which was then refined through consultation with NSW DPI team. The interview guide can be found in Supplementary Material.

A target of 20 interviews was set to achieve data saturation, with a plan to increase the number of interviews if required (Brod et al. 2009).

The interviews were conducted by the Charles Sturt University research team (LH) between 17th July and 21st August 2024, concurrently with the survey data collection. Purposive sampling of interview participants was undertaken, aiming for variability in demographic characteristics such as age, gender, and reason for boat ownership, with participants providing informed consent.

Audio recordings of the interviews were downloaded and transcribed into a Microsoft Word document using a professional transcription service. Interview data were uploaded to qualitative data analysis software NVivo (QSR International Pty Ltd. Version 1.6.1). Data were read with initial ideas noted. Responses were then coded to questions, with analysis of the content undertaken.

## Results

### Questionnaire Results

#### Questionnaire Participant Demographics

A total of 257 questionnaire responses were received, resulting in 192 usable responses. Participants’ responses were removed from the analysis if insufficient responses were provided, or if they reported owning trailered boats.

Table 1 describes the demographics of questionnaire respondents. Just under 90% of participants identified as male (*n* = 172), with the age group most represented being between 51 and 65 years of age (46.4%, *n* = 89). Most boats owned or managed by participants were between 10 and 20 m of length (60.4%, *n* = 116) and were moored in a private mooring (63.0%, *n* = 119). Over half of participants (53.9%, *n* = 103) reporting owning or managing the boat for more than 5 years. Note that henceforth, throughout this manuscript the terms ‘owner’ is used to describe those who own or manage a boat, unless specifically stated otherwise.

**Table 1:** Demographic information of owners/managers of small to medium boats moored or at berth in the NSW marine estate, 2024

	Number	Percentage
**Gender**	*192*	
Male	172	89.6
Female	15	7.8
Non-binary	3	1.6
Rather not say	2	1.0
**Age**	*192*	
18–34	8	4.2
35–50	45	23.4
51–65	89	46.4
66–80	44	22.9
Over 80	6	3.1
**Length of boat**	*192*	
Less than 5 m	3	1.6
5-less than 10	70	36.5
10-less than 20	116	60.4
20-less than 30	2	1.0
30 or more	1	0.5
**Mooring type**	*189*	
Marina	44	23.3
Boating/yacht club	13	6.9
Private mooring	119	63.0
Private jetty	7	3.7
Commercial Mooring	5	2.6
DPI mooring	1	0.5
**Main use**	*190*	
Cruising	115	60.5
Racing/sailing	32	16.8
Live	3	1.6
Fishing	29	15.3
Research/work	6	3.1
Diving/snorkelling	1	0.5
Project	1	0.5
Travel	3	1.6
**Owning duration**	*191*	
<12 months	17	8.9
1–5 years	71	37.2
>5 years	103	53.9
**Mostly used**	*192*	
Locally	159	82.8
Large distances across NSW	26	13.5
Interstate	7	3.6
International	0	
Seen Emergency Animal Disease (EAD) hotline number*	*192*	
No	169	88.0
Yes	23	12.0

^*^National toll-free telephone number that connects callers to the relevant state or territory animal health authority to report concerns about potential emergency animal diseases

Participants reported mainly using their boats for cruising (60.5%, *n* = 115), with most movements being local (82.8%, *n* = 159). When compared with Phase 1 respondents, the proportion of larger distance movements were higher among Phase 2 than Phase 1 respondents (17.2% and 7.7% respectively). The other main difference between the two populations was an increase in representation of Phase 2 participants who reported using their boats for cruising (60.5%, *n* = 115 compared with Phase 1–48.4%, n = 30), and a decrease in those that reported used their boats for racing (16.8%, n = 32, compared with Phase 1–32.3%, *n* = 20).

Of these respondents, the majority reported having not seen the EAD Hotline number (88.0%, *n* = 169). Of the respondents that reported seeing the EAD hotline number and supplied the location where they saw it, the largest number (*n* = 7) reported that they saw the number at a boat ramp. Four respondents reported seeing it at yacht/sailing clubs and three saw the EAD hotline number at marinas. Other locations included DPI offices, pamphlets associated with boat registration, and in association with antifouling products online.

#### Biosecurity Practices and Awareness

Respondents were asked to indicate their level of awareness of biosecurity generally, and specifically about the biosecurity risks associated with biofouling on their boats. Despite the majority of respondents reported knowing biofouling on boats can cause damage to the waterways (76.3%, *n* = 100), being aware of the need to clean their boats regularly (94.6%, *n* = 123) and that niche areas in their boat can develop biofouling (88.5%, *n* = 116), just over half (54.5%, *n* = 72) reported knowing that it was a requirement to report suspected marine pests and signs of aquatic diseases and a third reporting knowing about the General Biosecurity Duty (32.1%, *n* = 42). When compared with Phase 1 respondents, there was a slight increase in awareness of the impact of biofouling (62.3%, *n* = 33), while other responses were similar in frequency.

#### Self-reported Practice Change

Respondents reported whether they had made any changes to how they managed their boat in the last 18 months, including actions undertaken by others. The majority of respondents had not made any changes (Table 2), however, the biggest positive shift in behaviour was reported in checking boats for biofouling, with over a quarter of respondents reporting that they increased inspections in the last 18 months (26.9%, *n* = 36).

**Table 2:** Self-reported changes in frequency of cleaning practices in last 18 months by owners/managers of small to medium boats moored or at berth in the NSW marine estate, 2024

Cleaning related practices	Number	Percentage
**Checking boat for biofouling**	*134*	
Decreased frequency	4	3.0
No change in frequency	92	68.7
Increased frequency	36	26.9
Do not do this	2	1.5
**Applied antifouling to boat**	*134*	
Decreased frequency	8	6.0
No change in frequency	97	72.4
Increased frequency	21	15.7
Do not do this	8	6.0
**Slipped boat for biofouling cleaning**	*133*	
Decreased frequency	8	6.0
No change in frequency	97	72.9
Increased frequency	21	15.8
Do not do this	7	5.3
**Cleaned biofouling in water**	*130*	
Decreased frequency	4	3.1
No change in frequency	53	40.8
Increased frequency	33	25.4
Do not do this	40	30.8
**Use Bilge pump-out facility**	*134*	
Decreased frequency	4	3.0
No change in frequency	68	50.7
Increased frequency	4	3.0
Do not do this	58	43.3
**Checking equipment for biofouling**	*133*	
Decreased frequency	2	1.5
No change in frequency	97	72.9
Increased frequency	13	9.8
Do not do this	21	15.8

A quarter of respondents (25.4%, *n* = 33) also reported that they increased the frequency in which they cleaned biofouling in the water. Just under half of respondents reported that they did not use a bilge pump-out facility (43.3%, *n* = 58). This may mean either their boat does not require this or that they may pump out bilge into the water.

Respondents who indicated that they had made changes to how they managed their boats were asked to consider why those changes were made, with the majority reported that there was ‘no reason’ as to why they changed. Open comments in this section identified some drivers for the reported changes.

Of the respondents who reported an increased frequency of checking boat for biofouling, one respondent identified NSW DPI social media posts and one, information from a boat show as the reason behind their changed behaviour. Other reasons provided for increasing checking of biofouling included evidence of increased biofouling, whether visualised or discussed (22 respondents), and the need for better boat performance (2 respondents).

When reporting an increased frequency of antifouling application, the majority of respondents reported observed increased biofouling evidenced on their boats (*n* = 13), or a perceived failure of antifouling product (*n* = 10).

Twelve respondents identified that the reason they increased the slipping frequency of their boat was driven by the increase in observed biofouling.

One behaviour of concern that increased in frequency was cleaning boats in the water (10 respondents). One respondent identified NSW DPI social media posts as the reason they increased the frequency of cleaning their boat in the water, while three respondents reported that they cleaned in the water because the increase in biofouling meant that they could not wait for their annual slipping to clean.

#### Communication Preferences

Respondents were asked to identify in an open question who they would trust and go to with questions about unusual marine animals/plants on their boats. The DPI Biosecurity and Fisheries were the most trusted sources of information (84.0%, *n* = 63; 83.7%, *n* = 72, respectively), and were also reported as most used (67.1%, *n* = 49; 65.9%, *n* = 54, respectively) (Table 3).

**Table 3:** Trust and usage of information sources by owners/managers of small to medium boats that are moored or at berth in the NSW marine estate, 2024

Information source	Trust		Use	
	Number	Percentage	Number	Percentage
**DPI Biosecurity**	*75*		73	
Yes	63	84.0	49	67.1
No	12	16.0	24	32.9
**DPI Fisheries**	*86*		82	
Yes	72	83.7	54	65.9
No	14	16.3	28	34.1
**Port Authority of NSW**	*67*		62	
Yes	40	59.7	25	40.3
No	27	40.3	37	59.7
**Roads and Maritime Services**	*78*		72	
Yes	43	55.1	32	44.4
No	35	44.9	40	55.6
**National Parks**	*70*		66	
Yes	35	50.0	23	34.8
No	35	50.0	43	65.2
**EAD hotline**	65		64	
Yes	31	47.7	15	23.5
No	34	52.3	49	76.6
**Local Lands Services**	*66*		62	
Yes	25	37.9	10	16.1
No	41	62.1	52	83.9

Two respondents singled out government agencies as places they would not go for trusted information.

*“In my area I don’t think there is any authority I’d report to. I’ve noticed a significant increase in mussels attaching to the hull. I know that there is a mussel farm nearby and it’s new. Frankly I don’t believe raising this issue would serve any purpose as the government and business interests trump those of residents”*.

*“…as waterway users, we now view government orgs as outside. They have none of our interests at heart. None. Just more costings”*

Respondents were asked about which Facebook pages they would look at for posts about managing biofouling and marine pests and diseases. The majority of respondents (54.0%, *n* = 71) reported that they did not look at any of the suggested Facebook pages for biofouling information. The Facebook page for DPI Fisheries was the most popular site chosen (20.1%, *n* = 27) with the least selected being NSW DPI Biosecurity (14.2%, *n* = 19).

Of those respondents who identified other Facebook groups they used, dedicated boat groups, such as Sailing Australia and Boasting NSW, Boat Owners Association, and antifouling paint suppliers were mentioned. The government pages were also identified by one respondent as adverts that they did not look at and another reported that *“No one follows government pages in my circles”*.

#### Evaluation of Communication and Engagement Strategy

Respondents were asked if they remembered seeing images or videos posted by NSW DPI regarding marine biosecurity practices. The majority of respondents reported having access to Facebook but not seeing either the images or the videos (81.8%, *n* = 108; 90.2%, *n* = 119, respectively).

When asked to identify their preferred format of information, the majority identified electronic newsletters/fact sheets, with 30.6% identifying social media as a preferred avenue for information, and 16.4% reporting not wanting any information (Table 4).

**Table 4:** Preferred format of information reported by owners/managers of small to medium boats that are moored or at berth in the NSW marine estate, 2024

Preferred sources of information	Number	Percentage
Electronic newsletters/fact sheets	58	43.3
Websites	46	34.3
Social media	41	30.6
Apps	23	17.2
I do not want information	22	16.4
Print newsletters/fact sheets	17	12.7
In person workshops	6	4.5
One-on-one conversations	6	4.5

### Stakeholder Interviews

Expressions of interest in interview participation were received from 53 survey respondents. From this group, invitations to participate were sent to 38 potential participants, from which 18 interviews were conducted. Two of these interviews had two participants on the call.

All interviews were conducted via telephone, with the median duration of the interviews being 23 min (range 12–41 min).

The outcomes are presented collectively across the key areas of investigation, with individual quotations used to support the general discussion points.

#### Interview Participant Demographics

Table 5 provides a summary of the key demographic characteristics of the interview participants. The majority of interviewees reported using swing moorings, with five boat owners reporting using marinas.

**Table 5:** General demographics of interviewed owners/managers of small to medium boats that are moored or at berth in the NSW marine estate, 2024

ID	Gender	Age	Reason for boat ownership*	Duration of ownership and/or involvement with boating
01	Male	51–65	Cruising	>10 years
02	Male	66–80	Fishing	30 years
03	Male	66–80	Cruising	16 years
04	Male	51–65	Racing or sailing	>5 years*
05	Male	> 80	Cruising	50 years
06	Male	35–50	My work	>5 years*
07	Male	51–65	Cruising	>5 years*
08	Male	18–34	Fishing	>10 years
09	Male	>80	Cruising	50 years
10	Male	66–80	Cruising	20 years
11	Female	66–80	Racing or sailing	50 years
12	Male	51–65	Cruising	50 years
13	Male	51–65	Racing or sailing	>5 years*
14	Male	51–65	Cruising/My work	>5 years*
15	Female	66–80	Cruising	>5 years*
16	Female	35–50	Racing or sailing	40 years
17	Male	51–65	Racing or sailing/My work	>5 years*
18	Male	51–65	Cruising	>5 years*

#### Interactions with the NSW DPI Marine Biosecurity Communication and Engagement Interventions

All participants were asked if they had exposure to or had engaged with any NSW DPI targeted campaign’s activities with images used as prompts, with two reporting that they recalled having seen any of the posts (images and/or videos) and none recalling having any awareness of the activities undertaken.

Despite minimal self-reported engagement with the NSW DPI campaign, one positive area within the discussions was in relation to the level of interest that participants expressed in maintaining and protecting the health of the waterways, and was eloquently expressed by this interviewee.

*Really, I’d encourage you to start down with a supreme level of ignorance on our part, but a huge amount of willingness. So, I don’t know too many boaties who don’t love the environment that we get to use our boats in and would do anything to make it either better or to keep it safe. It’s just, no one knows what the hell it is. 14*

All participants were asked directly if they had ever received information on pests and diseases posing a risk to the waterways. Two participants indicated that they had been exposed to this type of information while boating in non NSW locations.

*And I noticed very much there signs everywhere (Canada) that said, “Please clean your boat,” before they’ve got kayaks, they’ve got runabouts and so on. “Please clean your boat before taking it away from this area so you don’t transfer environmental biosecurity devices, things from here to some other lake.” And I thought that was very good, but I haven’t seen that anywhere in Australia. 09*

Participants, did not consider that there was enough information on the risks that pests and diseases posed and how to manage them. Suggested methods of information distribution included through established networks including boat clubs, targeted social media groups, marinas and boats ramps etc., and through marine retailers.

Other specific suggestion to support engaging and useful communication about marine biosecurity management included appropriate focus, simple messaging, location specific advice and relevance.

*I think for recreational boaties, it’s got to the citizen in science care for the environment angle, like protect local air, protects your local waterway and spaces from invasive species. That’s got to be the angle, almost like the marine equivalent of land care. That is what will pique people’s interest, I think, in the recreational boating space.16*

*And I think you need to get your message across in about two and a half seconds. They glance and it either captures or it doesn’t. 15*

*Well, the first thing is you’re going to have to tell me why it’s relevant. Why is it relevant? If I went to a boat show, biosecurity, what do I need to know that for? So, you’d have to convince me why I need to be on the lookout for it. I can understand, for example, those fire ants, they reckon are coming down from Queensland, that were imported, I can see at a glance why that’s critical, but why do I need to be worried about bugs on my boat. 18*

The opportunity to participate in the current study was viewed by some participants as a catalyst for increasing their understanding of marine pests and diseases and how their behaviour could contribute to the biosecurity risk. A number of participants requested additional resources be sent to them following the interview and others offered themselves for further contact should follow up be required.

*Well, now that I’ve had this conversation with you and you’ve raised my awareness, notwithstanding the fact that I might, if there was some sort of resource to say how do you recognize unwanted guests or the like, well again, now that I’ve had the conversation with you will go, “Alrighty. Well, this is part of monitoring things. 01*

*And now we’ve had this conversation, that’s a bit remiss of me. I should go and follow DPI. 06*

This is a positive finding, showing that many boat owners within this group of participants did not appear to have attitudinal barriers in relation to behaviour change, particularly if such a change protects the waterways and, by extension, their experiences of them.

#### Barriers to and Awareness of Marine Biosecurity Risks

Barriers to engaging with marine biosecurity practices were identified during the interviews, and included the challenges keeping up with increased levels of biofouling

*But what we’ve noticed recently, the amount of shell is getting worse and worse. Port Hacking, they’re having terrible trouble with shell down there. 10*

*I aim to anti-foul every two years. But we are finding, anecdotally talking to boaties around here, the marine growth is becoming much more prolific and we’re getting more shell barnacles rather than just weed. 03*

The increased value of waterway or harbour side land was also mentioned as a factor contributing to the challenges of keeping boats clean of biofouling, with sites that had previously been used to slip boats, being sold for development. The risks associated with increased movement of boats further afield to affordable shipyards, or resorting to cleaning boats in the water were also reported.

*…the government’s not showing any interest in keeping waterfront land for wharf and bridge contractors and for boats to be moored and more importantly, boats to be slipped.18*

*So, they’re taking a boat from Sydney all the way to the Gold Coast or Brisbane because it’s easier and cheaper. So, you’ve got the boat growing gunk in Sydney, taking it to the Gold Coast or Moreton Bay. 07*

*But it does clearly open up risk that some people are going to go, “Oh, too high. Going to give this a scrape in the local waterway,”.01*

#### Communication and Information Networks

For most of those interviewed, there was a high level of uncertainty about how to identify an unusual a marine animal/plant.

*But the average guy, would he be really interested in biosecurity that he goes down to his boat once every fortnight goes down the harbour. Even if they saw it, they’d ignore it unless you could start telling them why they need to be concerned. But even then, I don’t think they’re going to be worried to the average guy. What would he be looking out for anyway? 18*

Government agencies such as NSW DPI and RMS were mentioned as organisations that would be contacted if something unusual was observed.

*Anyway, I would contact Fisheries. And if I couldn’t get onto somebody local, obviously I would go further afield. Or I’ll write … the other point of contact might be Maritime, Roles and Maritime, the local boating services officer who would then give me advice about where to go or what to do. 11*

*If it was some weird-coloured or something I’ve never seen growing on the boat before, I’d definitely tell someone about it…My first stop would probably be Fisheries. Yeah, that’s just my knowledge. Maybe EPA, I don’t know, but Fisheries have probably been my first stop. 07*

Lack of clarity about the roles and responsibilities of government agencies were also identified as challenges, including the difficulty in separating government support and extension from compliance activities, and perceptions of DPI being restricted to terrestrial biosecurity.

*And there is certainly, I think in the popular mind, DPI, very interested in primary industry that’s all about sheep and cattle and growing wheat and things and isn’t something that for me would come to mind at all as having a maritime interest. 03*

*I would query it, but I was thinking about this, I wouldn’t know who to contact really, because the maritime services here, there’s no interest in it at all. Council? I don’t know. I have no idea. 04*

*It’s hard, isn’t it? Because the people we work with in DPI, just because we know them personally, I know their motivation, but at the end of the day, there is a compliance arm to that as well and it’s hard to tread both roles.04*

*I think if you’re putting signs up or your source of information, if you really, if possible, should be separate to the bodies that are tasked with compliance. 15*

Overall, the interview analysis suggests that most participating boat owners consider healthy waterways as important but may not make the connection between this and their actions. The disconnect between waterway governing bodies and users is highlighted, as is the challenge for the governing bodies to balance compliance and information sharing.

## Discussion

This study evaluated interactions of owners/managers of small to medium recreational boats permanently moored or at berth in the NSW marine estate with NSW DPI communication and engagement interventions implemented between 2022–2024. The interventions were developed in Phase 1 of the project (unpublished results), and included employment of staff dedicated to biosecurity engagement, and a social media campaign aimed at increasing awareness of reporting unusual pests and biofouling management. While the survey and interviews suggested minimal direct engagement with the interventions that were implemented, important insights concerning ongoing and future approaches to strengthen biosecurity practices of boat owners in NSW were found.

State biosecurity and Fisheries organisations were identified as the most trusted government sources of information by participants. This is consistent with earlier Australian research that saw a greater preference for information from state government over other government sources (Stenekes et al. 2018). This study also provided qualitative evidence that while participating boat owners would consider contacting state organisations to get help with identifying unusual marine plants or animals, there is a general distrust of government information sources over industry or community sources of information. The DPI in NSW has a marine biosecurity compliance role and biosecurity officers may be responsible for investigations and initiating prosecutions for breaches of the Biosecurity Act (Australian Government 2023). This regulatory role will have consequences for the success or otherwise of DPI branded engagement strategies. Information sharing and development of trusting relationships can be more difficult if one party has obligations to report behaviours of the other parties (Davis et al. 2011; Engdahl and Lidskog 2014). Until this barrier is addressed or reflected in how engagement and communication material is designed and delivered, this barrier may continue to impact engagement by boat owners with biosecurity messaging.

The survey and interview findings about where people go for information regarding biofouling is consistent with previous research that suggests industry service providers such as slipways and marinas (Stenekes et al. 2018) are boat owners’ first choice, along with clubs and communities of boat users. The current study also highlighted the role of the internet, including social media as trusted and used sources of information, with interview data suggest that Facebook pages not associated with government branding, may be a better way to share biosecurity messages. These include antifouling paint suppliers and groups associated with different classes of boat use. The Facebook pages associated with government organisations, however, were not identified as being used by participants, with social media generally, and government sites specifically not necessarily being the communication channel of choice. If social media is to be used, it is recommended that nongovernmental pages are used, allowing for more trusted networks to disseminate information. Using a simple message that identifies the relevance the biosecurity practices being encouraged is also important. Good cleaning practices that support healthy waterways and communities should be the desired outcome, rather than compliance with the Biosecurity Act 2015 (Australian Government 2023). This includes dissemination of the disease hotline phone number, which could be marketed as a help tool, rather than a reporting one.

Opposition to government management strategies of the NSW marine estate were also highlighted, including their involvement in how waterway land is managed, with ramifications for access to yards where boats can be slipped for biosecure cleaning. Infrastructural barriers to behaviour change refer to those barriers outside the individuals’ control, such as access to slip yards. Golebie et al. (2023) suggested that when considering barriers to pro-environmental behaviours, perceptions of perceived barriers impacts the drivers of behavioural intentions. It can be argued that infrastructural barriers that lie outside the participants’ control may be perceived as greater barriers than those within boat owners’ control. In cases where barriers to behaviour adoption are perceived to be high, recent work has identified individual risk perception of an IAS incursion, sense of self efficacy and the effectiveness of the behaviour designed to mitigate the IAS risk may be significant predictors of protective behaviours (Golebie et al. 2023). These findings mean that these drivers need to be incorporated into intervention design.

Such structural barriers require policy changes, such as environmental planning that frees up land for boat yards, thus enabling physical opportunities for boat owners to make biosecure choices when cleaning their boats (Michie et al. 2011). Any further work aimed at supporting changes in how boat owners manage biofouling needs to acknowledge and address these barriers for any success in behaviour change programs (Golebie et al. 2021a).

Despite the lack of evidence to support any increase in biosecurity awareness and adoption of pro-environmental behaviours related to the DPI implemented communications and engagement strategies, there was a large amount of evidence from both the survey and interviews, that boat owners observe the marine environment closely, value it deeply and understand it well, including current waterway changes. The data strongly reflected that biofouling has significantly increased in the last 18 months, regardless of location, resulting in increased application of biofouling products and cleaning biofouling in the waterways. These consistent observations demonstrate that boat owners are not disconnected from the waterways and are observant and thinking critically about what they are seeing. Understanding biospheric values of waterway users, that are driven by concern for the environment and how these values may influence risk perception, and subsequently behaviour adoption, is important in considering the design, delivery and evaluation of pro-environmental behaviour messages (Golebie et al. 2021b).

The reported increase in biofouling could be an excellent avenue for strengthening boat owner involvement in biosecurity practices by harnessing the impact of IAS incursion into personal narratives including how individuals may be impacted (Golebie et al. 2021b). Boat owners are already looking for biofouling, and using this as a starting point, rather than the concept of biosecurity, would be an important approach to consider. Short messages based on relevance to and impact the end user and the marine environment could strengthen community involvement in waterway management. Using end users in a co-design approach to develop biosecurity messages and distribution avenues would be an important step forward (Sherring 2021).

The differences between those behaviours that are publicly evidence, such as talking about IAS and those behaviours that are private, such as individual cleaning practices, also require reflection (Golebie et al. 2021b). In the current study, some participants reported that by contributing to the project, they felt more responsible to consider their actions relating to the marine estate and to become more engaged with biosecurity related activities and online content. This suggest that the motivation of some participants to engage more deeply with biosecurity practices can be prompted by public discussion and social interaction. Relational interactions can provide opportunities for deeper reflection about action, that may prompt a shift in motivation, from an automatic, heuristic response, to one of reflection and planning (Michie et al. 2011).

## Conclusion

Despite limited evidence of engagement by owners/managers of boats that are permanently moored or at berth in NSW waters with the biosecurity interventions, the evaluation has highlighted the continued importance of relationships and understanding end user values and behavioural drivers in the design and dissemination information from government sources. Further work should be done to extend the communication approaches, including through media and in-person contact. These approaches should focus on information and expertise sharing, including impacts on the environment and individuals, and not on compliance and regulation. These findings extend beyond the marine estate and are consistent with research exploring terrestrial biosecurity both in Australia and globally.

## Supplementary information

Marine biosecurity evaluation interview questions

Marine Biosecurity Evaluation questionnaire

## Data Availability

Due to ethics constraints and privacy, the data used in these paper cannot be made publicly available.
