# Supplementary material for: Engagement by New South Wales Marine Estate Users with and Evaluation of Communication Approaches to Strengthen Biosecurity Practices
Source: Environ Manage. 2025 Jul 8;75(10):2614–24. doi: 10.1007/s00267-025-02221-2 (PMC12457554; doi:10.1007/s00267-025-02221-2)
Supplement: Supplementary file 1 — Marine biosecurity evaluation interview questions [file 267_2025_2221_MOESM1_ESM.pdf]

Marine biosecurity Evaluation Interview questions (for vessel owners)

1. Where did you see the survey?
2. Can you please tell me why do you own a vessel? How long have you owned a vessel for? Explain to us how you use your vessel (e.g., focus on movements, time in one location, how often they visit other waterways)
3. Do you clean your vessel regularly? How and where do you clean your vessel? Have you changed how and when you do this in the last 18 months (if yes, why and what?).
4. If you think about pest and disease risk to the waterways near you, what (diseases/behaviours/attitudes/groups) concerns you the most? Prompt – concern about future (climate change). With your own practices are there any changes that you would like to make to mitigate these but can't. Why?
5. If you had questions about an unusual marine animal/plant on your boat, who would you go to for information and advice? Why? Who wouldn't you go to? Why?
6. Have you ever received information on pests and diseases posing a risk to the waterways? If yes, from who? Was the information useful in helping you understanding of risk pathways, the importance of early detection and reporting of aquatic pests and diseases (practices - cleaning your boat in the water, pumping out bilge into water, not cleaning your boat at all etc)
7. Do you think there is enough information available on the risks and how to manage them? What are the gaps?
8. If exposed to the DPI- campaign – was the information helpful, if behaviour changed, why did you do this?
9. Is there anything else in relation to pest / animal biosecurity risks and practices that you would like to discuss that we have not covered in the previous questions?

Defined as biofouling (International Maritime Organization 2013), the accumulation of aquatic micro-organisms, plants and animals on recreational vessels have great potential to transport NIS through multiple water bodies, including marinas and conservation areas, and marine, fresh and brackish waters (Ferrario et al. 2017; Outinen et al. 2021).
