# Supplementary material for: Engagement by New South Wales Marine Estate Users with and Evaluation of Communication Approaches to Strengthen Biosecurity Practices
Source: Environ Manage. 2025 Jul 8;75(10):2614–24. doi: 10.1007/s00267-025-02221-2 (PMC12457554; doi:10.1007/s00267-025-02221-2)
Supplement: Supplementary file 2 — Marine Biosecurity Evaluation questionnaire [file 267_2025_2221_MOESM2_ESM.pdf]

Marine Biosecurity questionnaire

1. Do you own (or manage on behalf of an owner) a boat that STAYS moored or at a berth in coastal NSW waters for any period of time. (Yes/No - thank you for your interest. We do not require any further input from you.)
2. How old are you?
    - 18-34
    - 35-50
    - 51-65
    - 66-80
    - older than 80
3. What gender do you identify with?
    - Male
    - Female
    - Non-binary
    - Rather not say
4. Can you please tell us the length of your boat?
    - Less than 5m (16ft)
    - 5m (16ft) to less than 10m (32ft)
    - 10m (32ft) to less than 20m (65ft)
    - 20m (65ft) to less than 30M (98ft)
    - 30m (98ft) or more
5. When in NSW, where is your boat usually moored? Please tell us

    a. the nearest town /suburb, and b. the name of the waterway.
6. How is your boat usually moored. At a:
    - Marina
    - Boating or yacht club
    - Berth
    - Private Mooring
    - Private Jetty
    - Other
7. What do you MAINLY use your boat for?
    - Cruising
    - Racing or sailing
    - Travel
    - Fishing
    - Diving/snorkelling
    - Live aboard
    - My work

- Research
- Other (please specify)?

8. How long have you owned this boat for?
    - Less than 12 months
    - 1-5 years
    - More than 5 years
9. In the last 18 months, where have you MOSTLY use your boat?
    - Locally, mostly in the same waterway
    - Large distances across NSW
    - Interstate
    - Internationally
10. Have you made any changes in how you manage your boat in the last 18 months? This includes if others did these actions for you. (Increase frequency/decrease frequency/ no change/ do not undertake this action).
    - Checked your boat for biofouling (organisms that attach to submerged parts of a vessel or equipment)?
    - Had an antifouling paint coating applied or re-applied to the hull?
    - Slipped your boat for biofouling cleaning?
    - Had biofouling cleaned in the water?
    - Used a bilge water pump-out facility?
    - Checked/cleaned the equipment you use on your boat for biofouling, e.g. fishing equipment, anchors and chains, etc.?
    - Other
11. If you had questions about an unusual marine animal/plant on your boat, who would you trust to go to for information and advice? (DPI (Biosecurity), DPI (Fisheries), Roads and Maritime Services, Local Land Services, National Parks, Port Authority of NSW, Emergency Animal Disease hotline, other (please give details)) Please order from 1-3.
12. **Please select yes or no for the following statements.**
    - I know that biofouling on boats can cause damage to the health of the waterways.
    - I know that I need to clean biofouling from my boat regularly
    - I know that it is not just the hull but also other niche areas of my boat that can develop biofouling, such as engine cooling systems, bilge and ballast water tanks, the propeller and shaft and other water inlets and outlets
    - I know that everyone has to report suspected marine pests or signs of aquatic diseases.
    - I know about the General Biosecurity Duty as part of NSW *Biosecurity Act 2015*.
13. Do you remember seeing any of these images on Facebook over the last 18 months.
    - Yes
    - No, I have access to Facebook but did not see these images
    - No, I do not have access to Facebook
14. If yes or No to Q13 – On which Facebook pages would you look to find posts about managing biofouling and marine pests and diseases?

i. NSW DPI general
ii. DPI Fisheries
iii. NSW DPI biosecurity
iv. Maritime
v. Other
vi. None

15. How would you like to receive information about managing biofouling and marine pests and diseases
    - I do not want information
    - One-on-one conversations
    - In-person workshops
    - Print newsletters/fact sheets
    - Electronic newsletters/fact sheets
    - Smartphone applications (apps)
    - Social media
    - Websites
    - Other (please give details)
16. If yes to question 10 - What do you think caused the change you reported?

i. No reason I can think of
ii. DPI Social media posts
iii. Information from DPI from boat show
iv. Information from DPI from boat club event
v. Other
